# Supplementary material for: Prevalence of depression and its impact on quality of life in frontline otorhinolaryngology nurses during the COVID-19 pandemic in China
Source: PeerJ. 2021 Apr 20;9:e11037. doi: 10.7717/peerj.11037 (PMC8063879; doi:10.7717/peerj.11037)
Supplement: Supplemental Information 2 [file peerj-09-11037-s002.docx]

（中文版）

尊敬的耳鼻喉科护理同道们:

您好,本调查为中华护理学会精神卫生专委会、中华护理学会耳鼻喉科专委会和澳门大学联合发起，旨在了解抗击新型冠状病毒疫情中相关临床学科和科室护理工作者的精神心理健康状况，为今后开展相关职业健康教育、服务和护理培训提供依据。请您按照您的情况如实填写，问卷均以匿名形式提交，我们将严格保护您的信息，不会外泄您的个人资料。十分感谢您支持和参与！谢谢！

**基础信息表**

1. 问卷填写日期：____年____月____日
2. 您的年龄：________岁
3. 您的性别：□0=女 □1=男
4. 您的婚姻：□0=未婚 □1=已婚 □2=离异 □3=丧偶
5. 您的学历：□0=初中及以下 □1=高中/中专 □2=本科/大专 □3=硕士及以上
6. 居住状态：□0=独居 □1=与家人居住 □2=与朋友同事居住 □3=与其他人居住
7. 所在科室: □0=耳鼻喉科 □1=急诊科 □2=眼科 □3=其他科室
8. 职称： □0=初级 □1=中级以上
9. 您的从业年限：________年（整数，未满一年按一年进行纳入）
10. 是否参加过抗击SARS：□0=否 □1=是
11. 您的工作单位是：□0=三级医院 □1=初级医院
12. 目前实际工作所在科室：□0=门诊 □1=病房
13. 职务： 1＝倒班护士； 2＝行政班护士； 3＝护士长及以上
14. 您目前所在的省或直辖市是否已有确诊的新型冠状病毒肺炎疫情的发生？

1.有，10-99例

2.有，100-499例

3.有，500-999例

4.有，1000例以上

5.有，但不知道例数

1. 截至目前，是否有以下人员确诊为新冠肺炎
   □0=无 □1=家人 □2=同事或朋友 □3=负责的患者
2. 吸烟情况调查：

**1** 您过去曾经至少有一个月，每天至少吸烟1支？ 0=否； 1=是

**2** 您上一个月，每天至少吸烟1支？ 0=否； 1=是(不足1包) 2=是((1包或更多)

**3** 此次疫情爆发以来，您有无以前不吸烟，但是最近开始通过吸烟来减压？ 0=否； 1=是

1. PHQ-9抑郁症筛查量表

**请根据您最近一周的情况进行回答，如果一周内曾有波动，请以目前情况为准：**

|  | **完全不会** | **好几天** | **一半以上的天数** | **几乎每天** |
| --- | --- | --- | --- | --- |
| 1.做事时提不起劲或没有兴趣 | 0 | 1 | 2 | 3 |
| 2.感到心情低落、沮丧或绝望 | 0 | 1 | 2 | 3 |
| 3.入睡困难、睡不安稳或睡眠过多 | 0 | 1 | 2 | 3 |
| 4.感觉疲倦或没有活力 | 0 | 1 | 2 | 3 |
| 5.食欲不振或吃太多 | 0 | 1 | 2 | 3 |
| 6.觉得自己很糟，或觉得自己很失败。或让自己或家人失望 | 0 | 1 | 2 | 3 |
| 7.对事物专注有困难，如读报纸或看电视时不能集中注意力 | 0 | 1 | 2 | 3 |
| 8.动作或说话速度缓慢到别人已经觉察？或正好相反，烦躁或坐立不安、动来动去的情况更胜于平常 | 0 | 1 | 2 | 3 |
| 9.有不如死掉或用某种方式伤害自己的念头 | 0 | 1 | 2 | 3 |

1. 生活质量

1 您怎样评估您的生活质量？

1= 极不满意; 2=不满意; 3=没有满意或不满意; 4=很满意; 5=极满意

2 您满意自己目前的健康状况吗？

1= 极不满意; 2=不满意; 3=没有满意或不满意; 4=很满意; 5=极满意

（The English version）

Dear colleagues in otolaryngology,

Hello, this survey was jointly initiated by the Mental Health Committee of Chinese nursing association, and the Chinese nursing association otolaryngology branch and the University of Macau. It aims to understand the mental psychology of nursing workers in relevant clinical disciplines and departments in the fight against the novel coronavirus epidemic. The health status provides a basis for carrying out relevant occupational health education, services and nursing training in the future. Please fill in the questionnaires truthfully according to your situation. The questionnaires are submitted anonymously. We will strictly protect your information and will not leak your personal information. Thank you very much for your support and participation! Thank you!

**Basic information table**

1. Date of filling in the questionnaire：____ year ____ month ____ day
2. Age(years)：________
3. What is your gender? □0=female □1=male
4. What is your marital status? □0=unmarried □1=unmarried □2=divorced □3=widowed
5. What is the highest education you received? □0=junior middle school and below □1=high school/secondary school □2=Undergraduate/junior college □3=Master's degree and above
6. What is your living status? □0=living alone □1=living with family members □2=living with friends and colleagues □3=living with others
7. Department: □0=ENT □1=emergency department □2=ophthalmology □3= other departments
8. Professional title： □0=Junior □1=Senior
9. Years of Employment：________years (round number, less than one year will be included in one year)
10. Whether you have participated in the fight against SARS：□0=No □1=Yes
11. Your work unit is：□0=tertiary □1=primary
12. Departments where the actual work is currently located：□0=Outpatient □1=Inpatient department
13. What is your position? □0= Shift nurse □1=Administrative nurses □2= Head nurse and above
14. How about the number of COVID-19 confirmed cases in the province you lived in？

□0= Local COVID-19 cases 10-99

□1=Local COVID-19 cases 100-499

□2= Local COVID-19 cases 500-999

□3= Local COVID-19 cases ≥1000

□4= No certain

1. So far, have the following persons been confirmed as COVID-19
   □0=No □1= Family members □2= Colleagues or friends □3= Patients
2. **Smoking status：**

1 Have you ever smoked at least one cigarette a day for at least a month? □0=No □1= Yes

2 Have you smoked at least one cigarette a day in the past month? □0=No □1=Yes (less than 1 pack) □2=Yes ((1 pack or more)

3 Since this outbreak, have you never smoked before, but have you recently started smoking to reduce stress. □0=No； □1=Yes

1. **PATIENT HEALTH QUESTIONNAIRE (PHQ-9)**

Over the *last a week,* how often have you been bothered by any of the following problems?

|  | **Not at all** | **Several days** | **More than half the days** | **Nearly every day** |
| --- | --- | --- | --- | --- |
| 1. Little interest or pleasure in doing things | 0 | 1 | 2 | 3 |
| 2. Feeling down, depressed, or hopeless | 0 | 1 | 2 | 3 |
| 3. Trouble falling/staying asleep, sleeping too much | 0 | 1 | 2 | 3 |
| 4. Feeling tired or having little energy | 0 | 1 | 2 | 3 |
| 5. Poor appetite or overeating | 0 | 1 | 2 | 3 |
| 6. Feeling bad about yourself – or that you are a failure or have let yourself or your family down | 0 | 1 | 2 | 3 |
| 7. Trouble concentrating on things, such as reading the newspaper or watching television | 0 | 1 | 2 | 3 |
| 8. Moving or speaking so slowly that other people could have noticed. Or the opposite – being so fidgety or restless that you have been moving around a lot more than usual | 0 | 1 | 2 | 3 |
| 9. Thoughts that you would be better off dead, or of hurting yourself in some way. | 0 | 1 | 2 | 3 |

1. **Quality of life**

1 How would you rate your quality of life?

□0=Very poor □1= Poor □2= Neither poor nor good □3= Good □4= Very good

2 How satisfied are you with your health?

□0=Very dissatisfied □1=Dissatisfied □2=Neither satisfied nor dissatisfied □3= Satisfied □4=Very satisfied
